# Clinical status and future prospects of neoadjuvant immunotherapy for localized mismatch repair-deficient cancers: a review

**DOI:** 10.1097/JS9.0000000000001680

**Published:** 2024-05-20

**Authors:** Jian Li

**Affiliations:** Department of General Surgery, The Third Hospital of Mianyang, Sichuan Mental Health Center, Mianyang, Sichuan, People’s Republic of China

**Keywords:** immune checkpoint inhibitors, localized cancer, microsatellite instability, mismatch repair deficiency, neoadjuvant immunotherapy, nonoperative management

## Abstract

Frameshift mutations accumulate in cancers related to mismatch repair deficiency (dMMR), which has the potential to produce various neoantigens, representing a distinct subset of cancers that respond considerably to immunotherapy. In recent years, robust evidence has supported the first-line application of immunotherapy for patients with metastatic dMMR cancers, which provoked extensive investigations of the feasibility and efficacy of immunotherapy in up-front settings, including neoadjuvant therapy. Several completed trials with small sample sizes suggested that neoadjuvant immunotherapy can achieve an impressively high complete response rate, for the first time offering the potential of systemic therapy to cure cancer without the need for surgical resection. However, a difficult dilemma emerges: clinicians are now facing a selection between the standard of care with good evidence for proficient MMR but suboptimal for dMMR cancers and the emerging immunotherapy with promising results but only based on a limited number of patients with shorter duration of follow-up. This review aims to provide a comprehensive summary of the biological rationale and clinical status of neoadjuvant immunotherapy in patients with dMMR cancers. Furthermore, I elaborate on particular issues that must be taken into consideration for further advancement in the field.

## Introduction

Highlights

- In the past several years, several clinical trials on neoadjuvant immunotherapy for mismatch repair-deficient (dMMR) cancer have been published, offering systemic therapy to cure solid cancer without surgery for the first time.
- The comprehensive literature review of neoadjuvant immunotherapy for dMMR cancer is lacking, while this treatment paradigm may be a hot topic and will change the standard of therapy for localized dMMR cancer in coming years.
- This review summarizes the motivations and biological rationale for the application of neoadjuvant immunotherapy in patients with dMMR cancers, aiming to attract much more attention from stakeholders involved in the management of this specific cancer subtype.
- This review elaborates on further considerations for the application of neoadjuvant immunotherapy in dMMR cancers in clinical practice, which may be informative for future investigations.

Immunotherapy has revolutionized the treatment paradigm for advanced-stage cancers, providing an unprecedented survival benefit in selected patients^1,2^. However, only a minority of patients are expected to respond to immunotherapy^3^. Therefore, the application of neoadjuvant immunotherapy in localized cancers has attracted much attention in recent years, aiming to activate more solid anticancer immunity against existing neoantigens. In both localized and metastatic settings, an issue impeding the successful application of immunotherapy is the lack of perfect biomarkers for stratifying patients, as not every patient responds equally. Therefore, identifying reliable biomarkers to select patients who are likely to benefit from immunotherapy has attracted much attention since the initiation of the era of immunotherapy. Mismatch repair deficiency (dMMR), the predominant cause of microsatellite instability (MSI), is the first biomarker to perfectly predict the response of advanced cancers to immunotherapy^4^. dMMR boosts the accumulation of frameshift mutations, which can potentially produce a large amount of neoantigens^5^. In addition to the strong evidence indicating a poor response of dMMR cancers to chemotherapy, the impressive response rate to immunotherapy provoked extensive investigations of the feasibility and efficacy of immunotherapy in neoadjuvant settings (Tables 1, 2). These preliminary results suggest that immunotherapy can achieve a remarkably high complete response (CR) rate with a low incidence of severe adverse effects and rare primary or acquired resistance, providing strong support for exciting future directions, such as nonoperative management (NOM)^6–13^.

**Table 1:** Prospective studies of neoadjuvant immunotherapy in localized dMMR cancers.

Study	Phase	Patient (*n*)	Treatment	Duration (mo)	cCR	Surgical outcome (%)	NOM (*n*)	irAEs (%)	Follow-up	Survival
NEONIPIGA, 2022^6^	II	GC (16) GEJC (16)	Ipilimuma plus nivolumab	3+surgery+9	5/32 (15%)	R0:100 pCR:17/29 (58)	2	Any:75 Grades ≥3:19	14.9 (range 3.4–25.8)	No disease progression or recurrence; 1 treatment or cancer-unrelated deaths
INFINITY, 2023^7^	II	GC/GEJC (17)	Tremelimumab plus durvalumab	3+surgery	NR	R0:NR pCR:9/14 (64)	2	Any:NR Grades ≥3:17	NR	No disease progression or recurrence; 2 treatment or cancer-unrelated deaths
NICHE-1, 2020^8^	II	CC (20)	Ipilimuma plus nivolumab	0.5+surgery	NR	R0:100 pCR: 12/20 (60)	0	Grades <3:58 Grades ≥3:13	8.1 (IQR 5.6–13.8)	All patients were alive and disease-free
NICHE-2, 2022^9^	II	CC (107)	Ipilimuma plus nivolumab	0.5+surgery	NR	R0:100 pCR: 72/107 (67)	0	Grades <3: 57 Grades ≥3:4	13 (range 1–57)	No disease recurrence
PICC, 2022^10^	II	CRC (34)	Toripalimab with or without celecoxib	3+surgery+3	NR	R0: 100 pCR:26/34 (77)		Grades <3:59 Grades ≥3:3	14.9 (IQR 8.8–17.0)	All 34 patients were alive, no recurrence; 100% 1-year EFS, DFS, and OS
Cercek *et al.*, 2022^11^	II	RC (12)	Dostarlimab	6+observation	12/12 (100)	NA	12	Grades <3:75 Grades ≥3:0	12 (range 6–25)	No disease progression or recurrence; all 16 patients were alive
Ludford *et al.*, 2023^12^	II	CRC (27) Non-CRC (8)	Pembrolizumab	6 +surgery or 12+observation	10/33 (30%)	R0:NR pCR:10/15 (67)	18	Grades <3:37 Grades ≥3:6	9.5 (range 0–26)	1 treatment or cancer-unrelated deaths and 1 cancer-caused death
Chen *et al.*, 2023^13^	II	RC (16)	Sintilimab	Perioperative:3 or preoperative:3	11/16 (69)	R0:100 pCR:3/6 (50)	9	Overall:53 Grades ≥3:6	17.2 (IQR 8.2–28.5)	No recurrence, all patients were alive

CC, colon cancer; cCR, clinical complete response; CRC, colorectal cancer; dMMR, deficient mismatch repair; DFS, disease-free survival; EFS, event-free survival; GC, gastric cancer; GEJC, gastroesophageal junction cancer; IQR, interquartile range; irAEs, immunotherapy-related adverse events; NA, not applicable; NOM, nonoperative management; NR, not reported; OS, overall survival; pCR, pathological complete response; RC, rectal cancer.

**Table 2:** Ongoing studies of neoadjuvant immunotherapy in localized dMMR cancers.

NCT number	Phase	RCT	Tumor	Enrollment	Treatment	Planned NOM	Primary endpoint
NCT05729646	II	Yes	Gastric or GEJ cancer	90	Toripalimab plus CT or CT or toripalimab	No	MPR
NCT06250036	II	Yes	Gastric or GEJ cancer	50	Zimberelimab with or without domvanalimab	No	pCR
NCT06059495	II	No	Gastric or GEJ cancer	59	Dostarlimab	Yes	cCR
NCT05836584	II	Yes	Gastric or GEJ cancer	240	Atezolizumab with or without CT	No	EFS
NCT05994456	II	No	Gastric cancer	24	Toripalimab	No	pCR
NCT03257163	II	No	Gastric cancer	40	Pembrolizumab plus CT or RT	No	RFS
NCT05913570	II	No	CRC	22	Cadonilimab	No	pCR
NCT03926338	II	No	CRC	112	Toripalimab with or without celecoxib	No	EFS, pCR
NCT05371197	II	No	CRC	26	Envafolimab	No	pCR
NCT05116085	II	No	CRC	33	Tislelizumab	No	MPR
NCT05815290	II	No	CRC	50	Cadonilimab	Yes	cCR, pCR
NCT06262581	II	No	CRC	40	Tisleizumab	Yes	pCR
NCT04301557	II	No	CRC	25	Toripalimab plus CRT	No	pCR
NCT04715633	II	No	CRC	53	Camrelizumab plus apatinib	Yes	cCR
NCT05841134	II	No	CRC	25	Tislelizumab plus CT	No	pCR
NCT06002789	II	No	sMPCC	17	PD-1 monoclonal antibody	No	pCR
NCT06215677	II	No	Colon Cancer	18	Camrelizumab	No	R0 resection
NCT05239546	II	No	Colon cancer	25	Dostarlimab	Yes	MCR
NCT05890742	I	Yes	Colon cancer	100	Sintilimab with or without IBI310	No	pCR
NCT05855200	III	Yes	Colon cancer	711	Dostarlimab vs adjuvant CT or observation	No	EFS
NCT05961709	II	No	Colon cancer	50	Cemiplimab	Yes	EndoCR
NCT05662527	II	No	Colon cancer	85	Pembrolizumab	No	pCR
NCT04389151	II	No	Colon cancer	30	Toripalimab	No	MPS
NCT04643041	NR	No	Rectal cancer	47	PD-1 monoclonal antibody	Yes	1-year DFS
NCT04636008	Ib	No	Rectal cancer	20	Sintilimab plus HF-RT	No	Safety
NCT05732389	II	No	Rectal cancer	39	Nivolumab plus ipilimumab	No	cCR
NCT05723562	II	No	Rectal cancer	150	Dostarlimab	Yes	cCR
NCT05645094	NR	No	Rectal cancer	38	Envafolimab	Yes	cCR, pCR
NCT04357587	I	No	Rectal cancer	10	Pembrolizumab plus CRT	No	Safety
NCT04751370	II	No	Rectal cancer	31	Nivolumab plus ipilimumab plus CT	No	pCR
NCT05795244	II	No	Endometrial cancer	30	Nivolumab	No	pCR
NCT04262089	I	No	Uterine cancer	10	Pembrolizumab	No	Pathological response
NCT04556253	II	No	Gastric cancer and CRC	29	Cadonilimab	No	pCR
NCT04795661	II	No	Solid tumors	160	Pembrolizumab	No	pCR
NCT04082572	II	No	Solid tumors	35	Pembrolizumab	Yes	pCR
NCT04165772	II	No	Solid tumors	200	Dostarlimab or with CRT	Yes	pCR and cCR

cCR, clinical complete response; CRC, colorectal cancer; CRT, chemoradiotherapy; CT, chemotherapy; DFS, disease-free survival; EFS, event-free survival; EndoCR, endoscopic complete response; GEJ, gastroesophageal junction; HF-RT, hypofraction radiotherapy; MCP, major clinical response; MPR, major pathological response; NOM, nonoperative management; NR, not reported; pCR, pathological complete response; PD-1, programmed death-1; RCT, randomized controlled trial; RFS, relapse-free survival; RT, radiotherapy; sMPCC, simultaneous multiple primary colorectal cancer.

In this review, we briefly summarize the motivations for neoadjuvant immunotherapy in patients with dMMR cancers, followed by the underlying biological rationales. We further present preliminary clinical trial data on the feasibility, safety, and impressive efficacy of neoadjuvant immunotherapy in patients with dMMR cancer. Finally, we elaborate on particular issues that must be taken into consideration for further advancement in the field. Throughout the review, unless indicated, we will refer to the MSI-high status using the term dMMR to avoid confusion because of the high concordance between the two biomarkers.

## Motivations for neoadjuvant immunotherapy in patients with dMMR cancers

### Poor response to standard chemotherapy

A poor response to chemotherapy was demonstrated in both metastatic and localized dMMR cancers. For example, compared with their MMR-proficient (pMMR) counterparts, patients with metastatic dMMR cancers benefitted less from chemotherapy and had shorter overall survival (OS)^14–17^. Resectable dMMR cancers had better outcomes after surgical resection than did pMMR cancers, while perioperative chemotherapy dampened this survival benefit^18^. In the neoadjuvant setting, the FOxTROT trial reported that the majority of patients with dMMR colon cancer had little or no response to neoadjuvant chemotherapy^10^. A similar poor response was also observed in rectal cancer^19^. Currently, only guidelines for colon cancer do not support the routine use of adjuvant chemotherapy in patients with stage II dMMR disease; under other conditions, dMMR cancers are treated similarly to pMMR cancers, and a considerable number of patients experience disease relapse. Therefore, if chemotherapy is not the optimal choice for patients with dMMR cancer, a treatment paradigm change is needed to improve the cure rates of these patients, for whom promising outcomes have been demonstrated for immunotherapy.

### Perusing a strategy for nonoperative management

Currently, surgical resection alone or in combination with perioperative systemic therapy is the standard treatment for the majority of localized solid tumors. However, surgery and perioperative events, including anastomotic leakage, blood loss, infection and injury to adjacent organs, strongly interfere with recovery, negatively affect oncological outcomes, and are sometimes life-threatening^20^. Therefore, NOM has always been an attractive approach for these patients and has been demonstrated to be feasible and safe for patients with rectal cancer after chemoradiotherapy (CRT)^21,22^. In recent years, several prospective trials have shown a dramatically high CR rate but manageable adverse effects in patients with dMMR cancer treated by neoadjuvant immunotherapy, suggesting the potential of systemic therapy to cure cancer without the need for surgical resection for the first time (Table 1). The implications of NOM in dMMR cancers are substantial, as patients with dMMR cancer related to Lynch syndrome are young, in whom traditional therapies, especially radiation, can affect childbearing^23–25^. Therefore, using neoadjuvant immunotherapy to prevent radiotherapy and surgery may confer a particular benefit in young patients.

### Promising results from trials in patients with late-stage disease

The initial success of immunotherapy was achieved in tumors with a high mutational burden, such as dMMR cancers. Pembrolizumab was initially tested in the third-line setting, while an unprecedented objective response rate (ORR) was observed in patients with dMMR cancer^26^. In the second-line setting, nivolumab was given to patients with advanced dMMR colorectal cancer (CRC), and a 31% ORR was obtained, with 69% of patients having disease control for at least 3 months^27^. These promising results from late-line therapies led to the launching of the phase III KEYNOTE-177 trial recruiting patients with metastatic dMMR CRC without previous treatment^28,29^. Pembrolizumab significantly prolonged progression-free survival (PFS), and 83% of responders had a prolonged durable response^28,29^. Regarding metastatic dMMR cancers other than CRC, the KEYNOTE-158 study reported an ORR of 34.3%, with durability of response after immunotherapy lasting more than 30 months in more than 75% of patients who had an objective response^30^. These promising results in advanced dMMR cancers suggest that considerable efficacy of neoadjuvant immunotherapy can be achieved and maintained.

### More candidates in early-stage disease

A common challenge in the development and wide application of biomarker-directed agents is the lack of sufficient candidates. Generally, with the increase in stages, the prevalence of dMMR decreases dramatically. In colon cancer, the prevalence of dMMR was reported to be 15–20% in stage II diseases and 10–15% in stage III diseases, while dMMR was found in only 4–5% in metastatic diseases^15,31,32^. In gastric cancer, 86.8% of the MSI subtypes were diagnosed at stages I–III. Within each stage, the prevalence of the MSI subtype was reported to be 47% in stage I, 26% in stage II, 20% in stage III, and 12% in stage IV^23^. Real-world studies have also shown that more than 90% of dMMR gastric cancers were diagnosed in nonmetastatic patients^33,34^. Next-generation sequencing of 12 019 cancers revealed 8% of stages I–III cancers and 4% of stage IV cancers were dMMR, representing ~40 000 stages I–III diagnoses annually in the U.S. alone^35^. These findings suggest that there are broader application prospects for immunotherapy in patients with dMMR cancers at earlier stages.

## Biological rationale for neoadjuvant immunotherapy for dMMR cancers

**Figure 1:**
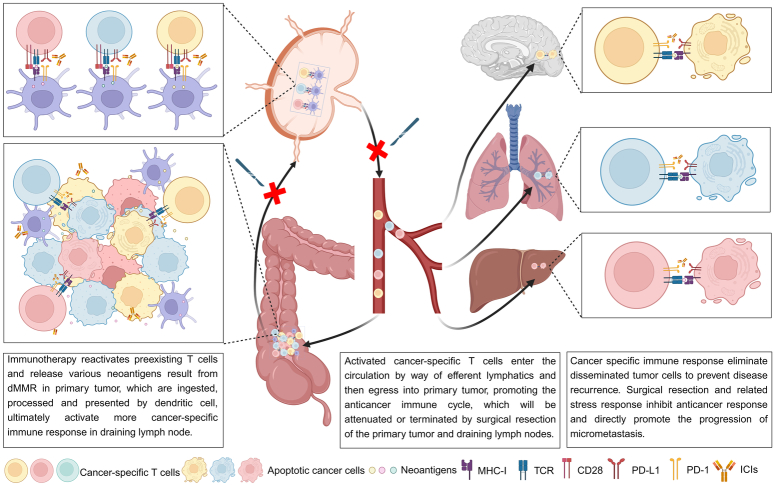
Biological rationale for neoadjuvant immunotherapy for dMMR cancers. Cancers related to dMMR produce a high number of neoantigens, which are recognized by specific T cells but suppressed by immune checkpoint molecules. Immunotherapy reactivates preexisting T cells and releases neoantigens, which are ingested, processed, and presented by dendritic cells, ultimately activating more cancer-specific immune responses in draining lymph nodes. Activated cancer-specific T cells enter the circulation via efferent lymphatics and then egress into primary tumors, promoting the anticancer immune cycle. They also patrol other organs to eliminate disseminated tumor cells. Surgical resection of the primary tumor and draining lymph nodes will interrupt the anticancer immune cycle induced by immunotherapy. dMMR, deficient mismatch repair; ICIs, immune checkpoint inhibitors; MHC-I, major histocompatibility complex-I; PD-1, programmed death-1; PD-L1, programmed death ligand-1; TCR, T cell receptor.

In recent years, neoadjuvant immunotherapy has been extensively investigated for localized solid tumors, and a more robust immune response against cancer has been demonstrated in neoadjuvant settings. Multiple theoretical advantages have been proposed to support the application of neoadjuvant immunotherapy and have been reviewed elsewhere^36,37^. Here, we briefly summarized these mechanisms as follows and focus on their associations with dMMR cancers (Fig. 1). First, anticancer immunity can be maximized by intact primary cancer and regional lymph nodes, which serve as sources of neoantigens and sites for the generation of effective and long-lasting anticancer immunity^36,38,39^. Theoretically, more neoantigens may be generated in dMMR cancers; keeping the primary tumor and draining lymph nodes intact may be more effective for anticancer immune activation than in general cancers. However, there are no direct data to support this hypothesis. Second, treatment-naive localized cancer has limited heterogeneity and a low tumor burden because tumor cells have not experienced extensive evolution or selection. High heterogeneity and tumor burden negatively affect immunotherapy outcomes^40,41^. Even in sporadic cases, dMMR is an early event in tumorigenesis, which results in extremely homogeneous dMMR status; however, discordance between primary tumors and metastases has been reported, indicating the application of immunotherapy in cancers at earlier stages^42^. Third, as discussed above, surgical resection, anesthesia, and perioperative events all seriously suppress anticancer immunity through stress responses^43,44^. Therefore, achieving an effective immune response may be easier before surgery. In addition, perioperative events extensively perturb physical status, making adjuvant immunotherapy less likely to be completed as planned than neoadjuvant immunotherapy^45^. Fourth, the majority of patients with cancer who died of distant metastasis developed from micrometastases, which cannot be eliminated by regional therapy involving surgery and radiation. Neoadjuvant immunotherapy has the potential to clear these micrometastases, providing the chance for a cure^46^. Fifth, biopsies and surgical specimens can be obtained during or after neoadjuvant immunotherapy, facilitating the development of potential predictive biomarkers and assessing patient response to guide clinical decision-making^36^, which holds particular significance in dMMR cancers, as the impressive pCR rate in these cancers provides a unique opportunity for NOM to avoid potential risks related to surgery.

## Clinical trials investigating neoadjuvant immunotherapy in patients with dMMR cancer

### Colorectal cancer

As colon cancer predominates in patients with dMMR tumors, current data on the efficacy of neoadjuvant immunotherapy are available mainly for colon cancer. In recent years, several studies have reported promising results, and NICHE-1 was the first one of prospective trials. In this trial, a combination of ipilimumab and nivolumab was administered. Among the evaluable patients, 100% of the 32 patients experienced a pathological response, with 97% a major pathological response (MPR) and 69% a pCR, which were significantly greater than those in the pMMR group and neoadjuvant chemotherapy group. At a median follow-up of 25 months, none of these patients experienced relapse^8,47^. In the following NICHE-2 study with the same immunotherapy regimen, a 99% pathological response was achieved in the 107 evaluable patients, consisting of 102 MPRs and 72 pCRs. No disease recurrence was observed at a median follow-up of 13 months^9^. Regarding localized rectal cancer, the single agent dostarlimab was tested in a single-arm phase II study. A total of 12 patients completed immunotherapy, and all of them achieved a clinical CR (cCR), avoiding further CRT or surgical resection. At a median follow-up of 12 months, no progression or recurrence was observed^11^. The safety and efficacy of another PD-1 antibody, sintilimab^13^, were also investigated in a single-arm, phase II study. Of the 16 patients evaluated, 6 underwent total mesorectal excision surgery, 3 of whom achieved a pCR, while the other 9 patients achieved a cCR and chose the NOM approach. No cases of death or relapse were reported after a median of 17.2 months of follow-up^13^. Other studies included patients with both dMMR colon and rectal cancer. For example, 34 patients with CRC in the PICC trial were randomized to receive toripalimab alone or in combination with celecoxib. R0 resection was achieved in all 34 patients, and pCR was observed in 65% of patients in the monotherapy group and 88% of patients in the combination group. After a median follow-up of 14.9 months, all patients were free of relapse, leading to a 100% 1-year event-free survival (EFS), disease-free survival (DFS), and OS^10^. In another trial, pembrolizumab was administered to 27 patients with localized CRCs. Among 14 patients who underwent surgical resection, the pCR rate was 79%. Thirteen patients elected to receive an additional 6 months of pembrolizumab without surgical resection. During the course of the trial and subsequent follow-up, progression events were only observed in 2 patients^12^.

### Gastroesophageal cancer

Given the dramatically improved survival in patients with metastatic dMMR gastroesophageal cancer (GEC) achieved by the addition of immunotherapy to traditional therapy, evaluating this approach in a neoadjuvant setting is natural. In a phase II cohort, patients with resectable GEC were randomized to receive perioperative chemotherapy alone or in combination with atezolizumab. A greater pCR rate was observed in the combination group (24% vs. 15%), and the difference was more pronounced in patients with dMMR (63% vs. 27%)^48^. Dual immune checkpoint inhibitors (ICIs) free of chemotherapy were also demonstrated to be unprecedentedly effective in treating dMMR GEC in two other prospective studies. In the NEONIPIGA trial, nivolumab, in combination with ipilimumab, was administered preoperatively. Of the 32 patients, 3 patients who experienced cCR refused surgical resection. All 29 patients who underwent surgical resection had an R0 resection, and 58.6% of them achieved pCR. At a median follow-up of 14.9 months, no patient had disease recurrence^6^. INFINITY is a single-arm trial evaluating the feasibility and efficacy of tremelimumab in combination with durvalumab as neoadjuvant therapy in patients with dMMR GEC. Among the 18 patients initially recruited, 2 achieved a cCR and refused surgery. Among 15 evaluable patients following surgery, 60% pCR and 80% MPR were observed. Except for 2 patients who died from other reasons after surgery, no cancer recurrence was observed. These promising results led to the continued recruitment of patients in cohort 2, in which NOM was evaluated after the completion of the same therapeutic regimen^7^.

### Other dMMR cancers

Currently, data on the efficacy of neoadjuvant immunotherapy in other dMMR cancers are scarce, and only a small number of case reports are available. A phase II trial included 7 patients with cancer other than CRC or GEC, including 2 with pancreatic cancer, 2 with duodenal cancer, 1 with ampullary cancer, 1 with endometrial cancer, and 1 with meningioma. Except for 1 patient with unresectable duodenal cancer who received only 1 cycle of pembrolizumab and died without surgery, 2 patients (1 ampullary and 1 duodenal cancer) achieved cCR and did not undergo surgical resection; the other 4 patients had partial regression or stable disease. Three patients (2 with pancreatic cancer and 1 with endometrial cancer) underwent surgical resection; although no pCR was achieved, at the database lock, all patients were alive^12^.

## Further considerations for application in clinical practice

### Surgical and organ-sparing considerations

Similar to the concerns in other neoadjuvant settings, preoperative immunotherapy has the potential to preclude patients with curable cancer from surgery because of disease progression^45^. However, the majority of studies on dMMR cancers have reported a very low incidence of disease progression and surgery preclusion, even with a longer duration of preoperative treatment^11–13^. The lower incidence of primary resistance in the neoadjuvant setting may be attributed to better physical status, relatively robust immune activity, low tumor burden, and limited heterogeneity^36^. Considering the small sample sizes in currently reported studies, primary resistance may be underestimated, warranting validation in larger populations. Elucidating the mechanisms of resistance and detecting resistance in a timely manner is important for preventing the inclusion of nonresponders. However, the mechanisms underlying the resistance of localized dMMR cancers to neoadjuvant immunotherapy have rarely been studied. Ludford *et al*.^12^ reported relatively greater rates of resistance and progression (17%), while none of these events occurred in patients with unresectable disease, indicating that local invasion does not necessarily indicate a greater likelihood of progression. The authors suggested that a higher infiltration of granulocytic cells in the tumor microenvironment and their proximity to CD8^+^ T cells may contribute to resistance to neoadjuvant pembrolizumab^12^.

NOM is the most exciting direction, as indicated by current findings from neoadjuvant immunotherapy in dMMR cancers. Although preliminary promising results have been reported, whether oncological outcomes are improved, compromised, or the same awaits validation in larger populations with longer-term follow-up. In addition, current radical resection of localized dMMR cancers has shown excellent long-term DFS^49^. Therefore, choosing NOM should carefully balance the risks of surgery and oncological outcomes. One issue is that the surgical challenge is not the same among all dMMR cancers. For example, radical colectomy is technically less complex, and postoperative organ function recovers well,^50^ making NOM less urgent in colon cancer, unless significantly improved oncological outcomes can be demonstrated in future studies. In contrast, because of anatomical constraints and complicated operation procedures, surgery for rectal cancer or GEC is associated with potential risks, which can be life-threatening^51,52^. In addition, secondary injury, malnutrition after gastrectomy, temporary or permanent stoma, and long-term adverse effects following standard perioperative pelvic radiation all negatively affect the quality of life^53–57^. Therefore, organ preservation was evaluated mainly in patients with dMMR rectal cancer and GEC. However, whether other cancer types can benefit from NOM after neoadjuvant immunotherapy or just trade known potential risks for other toxicities needs to be explored in well-designed, prospective, multicenter studies.

### Response assessment

Another challenge is how to make accurate response assessments and how to ensure patients with true cancer clearance receive NOM while monitoring disease recurrence conveniently and effectively. Accurate response determination is key for decision-making when NOM is planned, as misinterpretation leads to unnecessary surgical resection in patients who have achieved complete cancer clearance while putting patients with residual disease at risk of cancer progression^8,10,58^. Unfortunately, surgical specimen examination is the only modality with the ability to definitively ascertain whether residual tumor cells exist.

Currently, radiological examination remains the main method for assessing treatment response in the majority of cancers. However, previous studies reported that radiological response correlated weakly with pathological response^8,10,58^. Similar inconsistencies were also observed in neoadjuvant immunotherapy but with a distinct pattern. Previous studies have reported that the radiological response generally underestimates the pathological response in patients with solid cancers treated with neoadjuvant immunotherapy^59–62^. These underestimates were also found for dMMR cancers^10–12^. The paucity of cancer shrinkage may result from the infiltration of reactive immune cells rather than disease progression^63^. The interval between treatment initiation and response assessment is another cause, as a delayed response was consistently found in immunotherapy^64^. Therefore, 100% of the 12 consecutive patients achieved magnetic resonance imaging (MRI) CR after 6 months of immunotherapy, which is much longer than that reported in other studies^12^. On the other hand, utilizing radiological response as a surrogate of pathological response to inform NOM may have potential risks, as viable cancer cells were still present in biopsies from patients with a cCR by MRI^13^. Because of these limitations, endoscopic examination and biopsies were routinely performed to complement radiological examination, which indicated a correlation between endoscopic CR and subsequent pCR^12^. However, whether the stringent criteria for endoscopic CR in rectal cancer patients following CRT can also be applied to patients receiving immunotherapy is unclear, as patients with both stricturing and papillary mucosal changes following neoadjuvant immunotherapy have been subsequently demonstrated to have pCR^12,65^.

Therefore, no examination can perform accurate response assessments independently, and the integration of currently available or developing new methods is needed. For example, Cercek *et al*.^11^ employed digital examination, enteroscopy, and biopsy, MRI, and CT scans jointly to determine a clinical response and successfully ensured all patients to NOM. Several newly emerged assessment modalities, including positron emission tomography (PET)^11,66^, specimen-based biomarkers^8,11,12^, liquid biopsy^12,66^ and tumor organoids^8^, have also been evaluated, and their feasibility and potential to guide clinical decision-making were preliminarily demonstrated. However, sensitivity and specificity of these assessment modalities need to be improved and further validated in prospective studies.

### Study design and endpoint

As discussed above, although promising findings were reported, these studies were limited by small sample sizes, short follow-up periods, single-arm, and use of surrogate endpoints. However, considering the impressive efficacy of these treatments, these limitations are unlikely to affect the reliability of the evidence. It is difficult to perform randomized controlled trials (RCTs) with long-term follow-up periods to evaluate therapeutic outcomes from small groups of patients. Therefore, a specific biomarker-directed single-arm study has been developed, the findings of which are also believed to enable treatment selection. Nonetheless, the incidence of dMMR cancers is not low^67^, preventing the conduction of studies investigating neoadjuvant immunotherapy may be the perfect outcome achieved by traditional treatments. Considering the unprecedented high pCR rate and high potential for NOM, larger RCTs are encouraged to test neoadjuvant immunotherapy in patients with dMMR cancers.

In clinical oncological trials, survival outcomes remain the most commonly used endpoint. However, it is challenging to apply these endpoints to clinical trials, including those of patients with early-stage cancer, as a long period of time is needed to reach these endpoints. The excellent and durable efficacy of immunotherapy further decreases its feasibility. Therefore, surrogate endpoints have been adopted in studies of immunotherapy for early-stage cancers^68^. Clinical response and pathological response are widely assessed early endpoints in neoadjuvant trials^69,70^. Although excellent associations between pCR and survival outcomes, such as EFS, DFS, and OS, have been demonstrated in patients with various cancers after neoadjuvant immunotherapy,^71–74^ prospective evidence is lacking for dMMR cancers, and whether pCR can translate to survival benefit needs further follow-up. Theoretically, the response is likely to persist for a long time, as long-term durability of tumor regression was observed in advanced dMMR cancers treated with immunotherapy^29^. However, due to the limitations of insufficient follow-up, it is impossible to determine whether a cCR can successfully guide patients to undergo NOM. In addition, abundant data have demonstrated the ability of traditional treatment modalities to cure dMMR cancers at early stages, with acceptable risks and financial burdens. Are all patients prefer immunotherapy to other traditional modalities or are they satisfied with the outcomes following neoadjuvant immunotherapy? To answer these questions, patient-reported outcomes need to be thoroughly analyzed, which have not been involved in current studies.

### Heterogeneity in dMMR cancers

A notorious hallmark of cancer, making it difficult to be eliminated, is heterogeneity^41^. Although infrequent, heterogeneity has also been found in dMMR cancers^42,75^. However, determination of this heterogeneity is difficult before complete specimens are available, while its effects on neoadjuvant immunotherapy may be decisive, as less responsive but more invasive pMMR tumor cells may benefit from the elimination of dMMR tumor cells, contributing to disease progression.

Currently, neoadjuvant immunotherapy is mainly investigated for dMMR gastrointestinal cancers. The prevalence of dMMR varied significantly across cancer types, which may partially contribute to the various interests in neoadjuvant immunotherapy for different cancers. Using an MSI classifier to analyze 5930 patients with cancer, 30%, 19%, and 19% of endometrial, colon, and gastric cancer patients, respectively, were classified as MSI-high, lower but still detectable frequencies were observed in other cancer types^67^. Similar results were also reported by another study using a different approach^35^. However, whether the efficacy of neoadjuvant immunotherapy differs across cancer types is unclear. Less activity was observed in patients with dMMR pancreatic cancer in a prospective trial in which both patients with pancreatic cancer demonstrated adaptive progression and failed to achieve pCR^12^. Poor response of pancreatic cancer patients to immunotherapy was also observed in other trials^76^. Therefore, whether other dMMR cancers can obtain similar excellent efficacy as that in gastrointestinal cancers await the results from several ongoing clinical trials including various solid tumors (Table 2).

Furthermore, dMMR cancers can be sporadic and heritable, and the prevalence of alterations in the four genes in Lynch syndrome patients are not the same^77^. Theoretically, more homogeneity in MMR status can be observed in cancers related to Lynch syndrome than in sporadic cases. However, it is impossible to draw any conclusions regarding whether the response to immunotherapy differs between sporadic and inherited dMMR cancers. In a report by Cercek *et al*.^11^, 57% of the patients were found to have genomic alterations related to Lynch syndrome, while all patients responded completely to neoadjuvant immunotherapy, indicating that both types of dMMR cancers can benefit from neoadjuvant immunotherapy. Another challenge in patients with Lynch syndrome is the extent of surgical resection and surveillance following neoadjuvant immunotherapy. However, no survival advantage was demonstrated in a meta-analysis because of the potential risk of metachronous cancers, total or extended colectomy rather than segmental bowel resection, and more frequent surveillance was recommended for patients diagnosed with CRC related to Lynch syndrome^78,79^. However, considering the impressive outcomes achieved by neoadjuvant immunotherapy, whether less extensive resection and surveillance frequencies are feasible in these patients requires additional investigation.

### Duration of immunotherapy

The optimal duration of neoadjuvant immunotherapy in patients with localized dMMR cancers is difficult to determine. On the one hand, the time to reach a cCR varied significantly among patients, with the median time reported to be 5.2 months in a rectal cancer cohort^13^. A systemic review of dMMR CRC patients revealed that patients who received more than 1 month of neoadjuvant immunotherapy achieved a numerically greater percentage of CR than those who received treatment less than 1 month (75% vs. 65%)^80^. The longer duration requirement reflects that the response to immunotherapy has been shown to evolve over a period of months rather than weeks in dMMR cancers^35^. Therefore, only a 100% cCR was observed in the study in which patients were administered 6 months of immunotherapy^11^. On the other hand, the excellent efficacy of neoadjuvant immunotherapy can rapidly lead to a cCR in some patients^8,9,11,47^. For example, after the initiation of dostarlimab, the majority of biopsies exhibited an absence of viable tumors as early as 6 weeks, and 42% of patients achieved endoscopic CR at 3 months. Some patients even achieved pCR after only one cycle of immunotherapy^12^. Therefore, when a precise response assessment can be made, a personalized duration of neoadjuvant immunotherapy is superior to a fixed time period.

This paradigm may also be applied to postoperative therapy. Although postoperative adjuvant immunotherapy should be considered in patients with residual cancer cells, whether adjuvant therapy can improve survival in these patients is not yet known. In addition, whether the same approach is needed in patients who have achieved pCR is unknown. The same question is also unanswered in patients who were treated with NOM. Limited evidence suggests that immunotherapy should be continued. For patients pursuing NOM, an additional year of immunotherapy was recommended for a prospective trial. All 10 patients who completed the recommended cycles did not experience disease progression, while 2 of the 5 patients did not complete an additional 1 year of immunotherapy because of disease progression^12^.

### Monotherapy or combination therapy

In reported studies, although limited by small sample sizes, immunotherapy alone achieves impressive cCR and pCR, leading to the question of whether combination therapy necessary and, if necessary, what are the optimal combinations of neoadjuvant immunotherapy for dMMR cancers. As previous studies have suggested poor responses to chemotherapy, cytotoxic agents have rarely been incorporated into neoadjuvant immunotherapy regimens for dMMR cancers. CRT is the standard approach for neoadjuvant therapy in several cancer types. However, the response rate of dMMR cancers to CRT has been inconsistently reported^81,82^. An ongoing trial evaluating the efficacy of two preoperative doses of pembrolizumab and postoperative pembrolizumab plus CRT in localized dMMR gastric cancer will determine whether a synergistic effect can be observed between CRT and immunotherapy^83^. In addition, studies evaluating the efficacy of dual ICIs reported comparable response rates but increased the potential of adverse effects; thus, in combination with CTLA-4 inhibitors, they may add little response improvement to PD-1 blockade^6,8^. However, the benefits of the addition of CTLA-4 inhibitors cannot be exclusively negated because of their nonredundant effects^84^, while combination therapy may be administered for only a shorter duration than monotherapy.

An interesting finding reported in the PICC trial may direct future exploration for combination therapy^10^. In an exploratory analysis, compared with toripalimab alone, toripalimab, in combination with celecoxib, numerically improved the pCR rate^10^. Celecoxib is a selective cyclooxygenase-2 inhibitor that was shown to further activate T-cell-dependent immunity^85,86^. As dMMR cancers always harbor more neoantigens that can induce a strong anticancer response, the establishment of overt cancer may involve multiple immunosuppressive mechanisms. Therefore, synergistic effects may be obvious in combinations with other anticancer immune modulators rather than traditional therapies aimed at eliminating rapidly proliferating cells or increasing the antigenicity of cancer.

### Daily practice considerations

In the neoadjuvant era, especially for patient selection and response monitoring based on molecular characteristics, several daily practice considerations need to be noted. First, routine tumor tissue sampling is needed before or during neoadjuvant immunotherapy to direct clinical decision-making. However, some cancer types may present with dMMR, but it is difficult to obtain cancer tissues, such as pancreatic cancer and glioma^35,67^. Even for cancer types in which tumor samples can be obtained conveniently, repeated invasive procedures have potential risks. In these settings, liquid biopsies may provide important information. However, the turnaround time for results from liquid biopsies and other genetic tests needs improvement to ensure the timely commencement or transition of therapy^87^. Second, the reported trials incorporated various methods, including endoscopic examination, biopsy, CT scan, MRI, PET, ctDNA, and immunological feature profiling, to assess treatment response and to surveil disease recurrence, with more frequency than in general clinical practice^11,13^. In addition to their potential risks, uncovered by reimbursement for some costly examinations also impedes the promotion of neoadjuvant immunotherapy in patients with dMMR cancer and NOM.

In the future, technological improvement and the establishment of a centralized healthcare system will eventually foster the application of neoadjuvant immunotherapy in daily clinical practice.

## Conclusions

**Figure 2:**
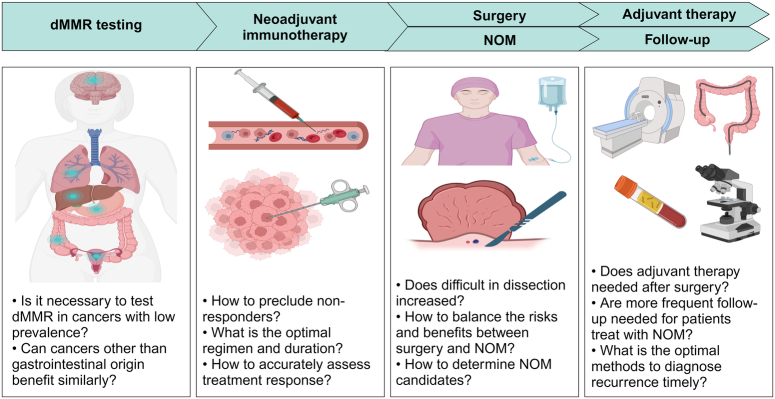
Further considerations for the application of neoadjuvant immunotherapy in clinical practice for dMMR cancers. Before routine application of neoadjuvant immunotherapy in clinical practice for dMMR cancers, from dMMR testing to follow-up, important questions must be answered in prospective studies with sufficient sample sizes, in which the balance between academic and community practices must also be considered. dMMR, deficient mismatch repair; NOM, nonoperative management.

Cancers related to dMMR represent a subset of cancers with distinct features. When at a localized stage, these cancers have a better prognosis and benefit poorly from chemotherapy. Before the advent of modern immunotherapy, localized dMMR cancers were routinely treated by surgery or similarly to their pMMR counterparts. In recent years, immunotherapy has achieved unprecedented success in treating metastatic dMMR cancers, which has provoked interest in investigating this approach at earlier stages. Similarly, neoadjuvant immunotherapy for dMMR cancers has achieved an impressive response rate, for the first time offering the potential of systemic therapy to cure cancer without the need for surgical resection. However, a difficult dilemma emerges: clinicians are now facing a selection between the standard of care with good evidence for pMMR but suboptimal for dMMR cancers and the emerging immunotherapy with promising results but only based on a limited number of patients with a shorter duration of follow-up. Many challenges await ahead, including organ-sparing considerations, accurate response assessment, optimal treatment duration and combination therapies, associations between short-term and long-term endpoints, and some daily practice considerations. Before routine clinical application, these questions must be answered in prospective studies with sufficient sample sizes, in which the balances between academic and community practices are also taken into consideration (Fig. 2). As evidence matures, an immunoablative approach may be established as a first and reasonable choice for patients with localized dMMR cancers.

## Ethics approval

Not applicable.

## Consent

Informed written consent is not necessary.

## Sources of funding

This work is supported by the Medical Research Project of The Third Hospital of Mianyang (202222) and the Scientific Research Projects of the Health Commission of Mianyang City (202223).

## Author contribution

J.L. designed the review, performed the selection of literature, drafted the manuscript, prepared the figures, and approved the final version of the manuscript.

## Conflicts of interest disclosure

The author declares no conflicts of interest.

## Research registration unique identifying number (UIN)

1. Name of the registry: not applicable.
2. Unique identifying number or registration ID: not applicable.
3. Hyperlink to your specific registration (must be publicly accessible and will be checked): not applicable.

## Guarantor

Jian Li.

## Data availability statement

The data that support the study findings are available upon reasonable request from the corresponding authors (Jian Li).

## Provenance and peer review

Not commissioned.

## Meeting presentation

None.
